# The conditions for connection—belonging as a health-promoting resource within higher education settings in Ireland

**DOI:** 10.1093/heapro/daag160

**Published:** 2026-10-27

**Authors:** Aisling McGrath, Maeve Doyle, Niamh O’Brien

**Affiliations:** Centre for Health Behaviour Research, Faculty of Health Sciences, South East Technological University (Waterford Campus), Cork Road, Waterford X91 K0EK, Ireland; SocialCORE, Department of Humanities, South East Technological University (Carlow Campus), Kilkenny Road, Carlow R93 V960, Ireland; Department of Education, Faculty of Education and Lifelong Learning, South East Technological University, Waterford Campus, Cork Road, Waterford X91 K0EK, Ireland

**Keywords:** student wellbeing, belonging, loneliness, social connectedness, higher education, health-promoting universities, healthy campus

## Abstract

Student wellbeing has become an increasing concern within higher education, with evidence highlighting the importance of social connection and belonging for mental health. Guided by settings-based health promotion principles, this study examined relationships between wellbeing, belonging, loneliness, and social connectedness among university students. A cross-sectional survey was conducted among students attending a multi-campus technological university in Ireland (*N* = 409). Participants completed validated measures of mental wellbeing (Warwick–Edinburgh Mental Wellbeing Scale), university belonging, loneliness (UCLA-3), and social connectedness, alongside demographic, and contextual questions. Data were analysed using descriptive statistics, correlations, group comparisons, hierarchical regression, and mediation analysis. Low mental wellbeing was reported by 44.1% of students, while 55.4% experienced elevated loneliness. Mental wellbeing was positively associated with university belonging (*r* = .51) and social connectedness (*r* = .45) and negatively associated with loneliness (*r* = −.41) (all *P* < .001). University belonging emerged as the strongest predictor of wellbeing (*β* = .325, *P* < .001), explaining variance beyond demographic and contextual factors. A significant indirect association between loneliness and wellbeing through university belonging was also observed. Female, sexual minority, first-year students, and students whose commuting affected participation reported poorer outcomes on selected measures. Findings highlight the central role of belonging in student wellbeing and suggest that belonging functions as an important health-promoting resource within higher education. Consistent with Healthy Campus and Health Promoting University approaches, universities should move beyond individualized wellbeing interventions and create social, organizational, and environmental conditions that foster equitable connection, participation, and belonging for increasingly diverse student populations.

Contribution to Health Promotion

- Nearly, half of students reported low wellbeing and over half reported elevated loneliness, highlighting social connection as a significant health promotion concern within higher education.
- University belonging emerged as the strongest predictor of wellbeing and partially mediated the relationship between loneliness and wellbeing.
- Findings support a shift from individualized approaches to student wellbeing towards settings-based approaches that foster connection, participation, and inclusion.
- Belonging may function as a health-promoting resource across diverse student populations, although opportunities to experience belonging may not be equally distributed.
- The study reinforces the importance of Healthy Campus and Health Promoting University approaches that create environments where belonging can flourish.

## Introduction

Health is created and lived within the settings of everyday life (WHO 1995) and since the publication of the Ottawa Charter for Health Promotion (WHO 1986), settings-based approaches have emphasized that shaping of health goes beyond individual behaviours and characteristics but also by social, organizational, and environmental conditions in which people live, learn, work, and interact (Dooris and Doherty 2010). Educational institutions represent one of the most important settings for promoting health and wellbeing, given their extensive reach across the life course and their capacity to influence social relationships, participation, identity development, and access to resources (Dooris 2009, HEA 2021, WHO 2015, Pulimeno *et al*. 2020).

This perspective has informed the development of Healthy Campus and Health Promoting University approaches, which recognize that student and staff wellbeing is shaped not only by individual support services but also by institutional culture, policies, physical environments, teaching practices, and opportunities for participation (WHO 2015, HEA 2021, Muraleedharan *et al*. 2025). The Okanagan Charter advanced this agenda by calling on higher education institutions to embed health into all aspects of campus culture and to lead health promotion action locally and globally (WHO 2015). More recently, the Limerick Framework for Action has built on the Okanagan Charter by providing an implementation-oriented roadmap for advancing health-promoting campuses through the interconnected pillars of People, Places, and Planet (International Health Promoting Campuses Network and University of Limerick 2025). Its emphasis on whole-system action, active participation and partnership is particularly relevant to understanding how higher education institutions can create conditions that support health, wellbeing and inclusion. These frameworks position universities as more than places of learning, but as settings that actively creates the conditions that enable, or constrain, health, wellbeing, inclusion, and flourishing (Zhao *et al*. 2025). From this perspective, students’ sense of connection and belonging should not be understood as incidental features of university life, but as potentially important outcomes of the social and organizational conditions created within higher education settings. This was particularly noteworthy in the Higher Education Authority’s review of the Irish National Framework for Student Success where wellbeing was noted as foundational element rather than an add-on (HEA 2025).

Despite growing international commitment to Healthy Campus and Health Promoting University approaches, concerns regarding student wellbeing continue to increase (Mahon *et al*. 2024, Li *et al*. 2025, Muraleedharan *et al*. 2025). While higher education has traditionally been associated with young adults navigating the transition to adulthood, contemporary universities serve increasingly diverse student populations (Thompson *et al*. 2021, Mahon *et al*. 2024). Alongside traditional undergraduate students, institutions now support mature learners, international students, students from diverse cultural and linguistic backgrounds, students balancing employment and study, students with caring responsibilities, and those living with disabilities or long-term health conditions (Mahon *et al*. 2024). Consequently, students enter higher education with varied experiences, resources, and opportunities for engagement. This diversity has important implications for health promotion because access to the social, academic and informal spaces through which connection is developed may not be equally distributed across the student population or settings where universities are multi-campus.

For many students, university represents a period of significant transition characterized by academic demands, identity development, changing social relationships, financial pressures, and new forms of independence (Griffin *et al*. 2025). However, these experiences are not uniform. Factors such as commuting, employment commitments, financial circumstances, family responsibilities, and social identities may influence students’ capacity to participate fully in university life and access the social and developmental benefits associated with higher education (Tett *et al*. 2025). National and international evidence indicates rising levels of psychological distress among university students, including increasing experiences of anxiety, stress, loneliness, and social isolation (Mahon *et al*. 2024, Akram *et al*. 2025, O'Donnell *et al*. 2025, Tian *et al*. 2025, McGrath *et al*. 2026). In Ireland, recent national research has similarly highlighted significant mental health challenges among third-level students, particularly within technological universities (Mahon *et al*. 2024, O'Donnell *et al*. 2025, Oti *et al*. 2025, McGrath *et al*. 2026). These trends have prompted increasing recognition that universities have an important role not only in responding to mental health difficulties, but also in creating the conditions that support wellbeing, connection, participation, and flourishing for increasingly diverse young student populations (HEA 2021, Li *et al*. 2025, Muraleedharan *et al*. 2025, Dooley *et al*. 2026).

From a health promotion perspective, wellbeing extends beyond the absence of mental illness (WHO 1995). This broader understanding is important because student wellbeing in higher education is often discussed through the language of psychological distress, help-seeking, support service demand, and individual resilience. While these concerns are important, they can obscure the social, relational, and institutional conditions through which wellbeing is produced. Rather than focusing solely on symptoms of distress, contemporary understandings of wellbeing emphasize positive functioning, meaningful participation, supportive relationships, purpose, and the ability to thrive within everyday environments (Dooris and Doherty 2010, Newton *et al*. 2016, Gautam *et al*. 2024). Wellbeing is therefore understood as a dynamic outcome arising from interactions between individuals and the social, cultural, and organizational contexts in which they are embedded (Diehl *et al*. 2018, Miyamoto and Ryff 2022). Within higher education, this perspective shifts attention from individual resilience alone towards the broader conditions that enable students to engage, participate, and flourish within and beyond university. Consequently, understanding student wellbeing requires consideration not only of psychological factors, but also of the social experiences and institutional environments that shape students’ everyday lives.

Among the factors increasingly recognized as central to student wellbeing, belonging has been identified as a particularly important construct within higher education research (Worsley *et al*. 2021, Hossain *et al*. 2023, Keshoofy *et al*. 2023, Tett *et al*. 2025), broadly defined, belonging refers to an individual's sense of being accepted, valued, respected, and included within a community (Strayhorn 2019). Within university settings, belonging reflects the extent to which students perceive themselves as legitimate and connected members of the academic and social environment (Thomas 2012). Students who experience a strong sense of belonging are more likely to engage academically, persist in their studies, participate in campus life, and report higher levels of wellbeing (Lowe 2023, Strayhorn 2019). Conversely, students who feel disconnected from their institution are at greater risk of disengagement, loneliness, poorer academic outcomes, and psychological distress.

The importance of belonging has been widely documented across higher education research. Tinto's theory of student integration proposes that students are more likely to persist and succeed when they become academically and socially integrated within university life (Tinto 1993). Similarly, Strayhorn conceptualizes belonging as a fundamental human need and argues that it represents a key determinant of student success, particularly for students from historically under-represented groups (Strayhorn 2019). Other research has also highlighted the role of belonging in shaping students’ confidence, motivation, identity development, and overall university experience (Schlossberg 1989, Hausmann *et al*. 2007, Gopalan and Brady 2020, Pedler *et al*. 2022). This suggests that belonging is not just a desirable aspect of student life, but a critical resource that supports both educational and wellbeing outcomes.

Closely related to belonging are the concepts of loneliness and social connectedness, constructs are increasingly recognized as important determinants of health and wellbeing across the life course (Holt-Lunstad *et al*. 2015, McClelland *et al*. 2020). Among university students, experiences of loneliness have been associated with poorer mental health, lower academic engagement, reduced life satisfaction, and increased risk of withdrawal from studies (Worsley *et al*. 2021, Dobos *et al*. 2024). In contrast, stronger social connectedness has consistently been associated with improved wellbeing, resilience, and positive educational outcomes (Allen *et al*. 2021, Pedler *et al*. 2022), suggesting that the quality of students’ social relationships and their sense of connection to others may play an important role in shaping wellbeing within higher education settings. From a health promotion perspective, these experiences should not be understood solely as individual psychological states, but as experiences shaped by the social, organizational and environmental conditions of university life.

Despite the growing evidence linking belonging, loneliness, connectedness, and wellbeing, these constructs are often viewed primarily as interpersonal or psychological phenomena arising from individual relationships, social skills, or students’ capacity to integrate into university life (Tinto 1993, Strayhorn 2019, Allen *et al*. 2021, Holt-Lunstad 2024). Comparatively less attention has been paid to the role of the university itself in shaping opportunities for connection and belonging. From a settings-based health promotion perspective, belonging may be understood not only as an individual experience but also as an outcome influenced by the social, organizational, and environmental conditions of the institution (WHO 2015, Newton *et al*. 2016, Allen *et al*. 2021). Factors such as campus culture, curriculum design, opportunities for participation, physical and virtual learning environments, institutional policies, support services, commuting patterns, employment commitments, and broader socioeconomic circumstances may all influence students’ capacity to engage with university life and develop meaningful social connections (HEA 2021). Participation, however, is not equally available to all students, and belonging may depend on access to the formal and informal spaces where relationships, recognition, and community are built (Tett *et al*. 2025). In this sense, belonging may be viewed as both relational and structurally conditioned, emerging through interactions between students and the environments in which they study.

This perspective aligns closely with Healthy Campus and Health Promoting University approaches, which emphasize the creation of supportive environments that enable participation, inclusion, equity, and wellbeing (HEA 2021). Rather than viewing loneliness, disconnection, or poor wellbeing solely as individual problems requiring individual-level solutions, health promotion approaches encourage examination of the wider conditions that facilitate or constrain opportunities for connection and belonging. Belonging should therefore not be understood simply as something students bring with them or develop privately, but as an experience shaped by the extent to which the university operates as a supportive, participatory, and health-promoting social system. Understanding these conditions is increasingly important as higher education institutions seek to address rising concerns regarding student wellbeing while supporting increasingly diverse student populations. In this framing, belonging is not simply an outcome of student adjustment to university life; it may also be understood as a salutogenic indicator of how health promoting the university environment is (Newton *et al*. 2016).

Although a substantial body of research has demonstrated associations between wellbeing, belonging, loneliness, and social connectedness in higher education these constructs are often examined independently or framed primarily as individual psychological experiences, with less attention paid to how they interact within broader institutional environments (Strayhorn 2019, Allen *et al*. 2021). Moreover, while universities are increasingly adopting Healthy Campus and Health Promoting University approaches, belonging has received comparatively limited attention as an explicit health-promoting resource through which higher education settings may influence student wellbeing (Dooris and Doherty 2010, WHO 2015, HEA 2021, Pani *et al*. 2025). Existing research has largely examined belonging in relation to student engagement, retention and academic success, with less consideration of its potential role as a pathway linking institutional environments to health and wellbeing outcomes (Thomas 2012, Strayhorn 2019). Although previous research has identified several demographic and contextual factors associated with student wellbeing and belonging, there remains a need to better understand how higher education environments create, facilitate, or constrain opportunities for connection, participation, and belonging among increasingly diverse student populations. Addressing these gaps is important for informing whole-institution approaches to wellbeing that move beyond individual-level interventions and consider the wider social and organizational conditions that support student flourishing. Understanding how belonging, loneliness, and social connectedness relate to wellbeing may help universities identify modifiable aspects of the campus environment that can strengthen student health and wellbeing.

The aim of this study was to examine mental wellbeing, university belonging, loneliness, and social connectedness among students attending a multi-campus technological university in Ireland. Drawing on a settings-based health promotion perspective, the study sought to explore both the relationships between these constructs and the demographic and contextual factors associated with variation in student experiences.

## Methods

This study employed a cross-sectional survey design to examine mental wellbeing, university belonging, loneliness, and social connectedness among students attending a multi-campus technological university in Ireland. The study was informed by a settings-based health promotion perspective, which recognizes that health and wellbeing are shaped by the social, organizational, and environmental conditions within which people live, learn, and interact. Accordingly, the survey sought to examine not only students’ wellbeing outcomes but also factors associated with opportunities for connection, participation, and belonging within the university setting.

Data were collected through an anonymous online questionnaire incorporating validated measures of mental wellbeing, university belonging, loneliness, and social connectedness, alongside demographic and contextual information relating to students’ experiences of university life (see Table 1). The study formed part of a broader institutional initiative examining student wellbeing and belonging across the university.

**Table 1:** Summary of measures included in the study.

Construct	Measure	Description	Response scale	Interpretation
Demographic and contextual characteristics	Study-specific items	Age, gender identity, sexual orientation, ethnicity, international student status, first language, disability status, campus, programme and year of study, financial support, employment status, working hours, travel mode, commute duration, and perceived impact of commuting on participation.	Variable formats	Descriptive and explanatory variables
Mental wellbeing	Warwick–Edinburgh Mental Wellbeing Scale (WEMWBS) (Tennant *et al*. 2007)	14-item measure assessing positive mental wellbeing, including positive affect, interpersonal relationships, and psychological functioning.	5-point Likert scale (1 = none of the time to 5 = all of the time)	Higher scores indicate greater mental wellbeing
University belonging	Adapted Psychological Sense of School Membership Scale (PSSM) (Goodenow 1993, Alkan 2016)	18-item measure assessing students’ perceptions of acceptance, respect, inclusion, and support within the university environment.	5-point Likert scale (1 = not at all true to 5 = completely true)	Higher scores indicate stronger university belonging
Social connectedness	Social Connectedness Scale (Lee *et al*. 2001)	20-item measure assessing feelings of interpersonal closeness, connection, and integration with others.	6-point Likert scale (1 = strongly disagree to 6 = strongly agree)	Higher scores indicate stronger social connectedness
Loneliness	Three-item UCLA Loneliness Scale (Hughes *et al*. 2004)	Brief measure assessing subjective feelings of loneliness, lack of companionship, feeling left out, and social isolation.	3-point Likert scale (1 = hardly ever to 3 = often)	Higher scores indicate greater loneliness

The study was at a multi-campus higher education institution located in the south-east of Ireland. The university serves a diverse student population across several campuses and offers programmes spanning health sciences, social sciences, business, law, engineering, science, computing, education, and the arts. As a technological university it reflects many of the characteristics associated with contemporary higher education, including a diverse student body comprising traditional school-leaver entrants, mature students, international students, commuter students, and students balancing employment and study. Given the distributed nature of the university and variation in campus size, location, and student composition, the setting provided an opportunity to examine wellbeing, belonging, and social connectedness across a range of higher education contexts.

### Participants and recruitment

Students enrolled at the university were invited to participate in an anonymous online survey. Informed consent was obtained electronically from all participants prior to participation. At the beginning of the online survey, participants were presented with study information and a consent statement and were required to indicate their consent via a mandatory response before proceeding to the survey questions. The survey was open to all students across all campuses, disciplines, and years of study, with a particular emphasis on the recruitment of undergraduate students. A non-probability convenience sampling approach was used. Recruitment was facilitated through Students’ Union social media channels and networks, as well as email distribution by academic staff and posters in student common areas. The survey remained open throughout November and December 2025. Participation was voluntary, and informed consent was obtained electronically before participants accessed the questionnaire. The study formed part of a broader institutional initiative examining student wellbeing and belonging across the university.

Data were collected through an online questionnaire incorporating validated measures of mental wellbeing, university belonging, loneliness, and social connectedness, alongside demographic and contextual information relating to students’ experiences of university life. Demographic and contextual items included age, gender identity, sexual orientation, ethnicity, international student status, first language, disability status, campus, programme and year of study, financial support, employment status, working hours, travel mode, commute duration, and perceived impact of commuting on participation. A total of 481 survey responses were received. Following data screening, the final analytic dataset comprised 409 students.

### Data analysis

Data were analysed using IBM SPSS Statistics (Version 29). Prior to analysis, the dataset was screened for completeness, missing data, and eligibility. Scale scores were calculated in accordance with published scoring guidance for each measure, with higher scores indicating greater levels of the relevant construct. Established Warwick–Edinburgh Mental Wellbeing Scale (WEMWBS) cut-off scores were used to categorize mental wellbeing, and recommended thresholds for the Three-Item UCLA Loneliness Scale were used to classify loneliness. University belonging and social connectedness were analysed as continuous variables, with higher scores indicating stronger belonging and greater social connectedness. Descriptive statistics, including frequencies, percentages, means, and standard deviations, were calculated to summarize participant characteristics and levels of mental wellbeing, university belonging, loneliness, and social connectedness. Pearson correlation coefficients were used to examine associations between wellbeing, belonging, loneliness, and social connectedness. Independent samples *t*-tests and one-way analyses of variance (ANOVA) were conducted to examine differences in outcomes across demographic and contextual characteristics. Where significant omnibus effects were identified, Bonferroni *post hoc* tests were performed to determine specific group differences. To examine predictors of mental wellbeing, hierarchical multiple regression analysis was conducted. Demographic variables were entered in the first block, contextual factors in the second block, and psychosocial variables (belonging, loneliness, and social connectedness) in the final block. Standardized beta coefficients (*β*), explained variance (*R*^2^), and significance levels were examined to determine the relative contribution of each predictor. Finally, mediation analysis was conducted using PROCESS Macro Version 5 (Hayes 2022) to examine the indirect association between loneliness and mental wellbeing through university belonging. Bootstrap confidence intervals were generated using 5000 resamples. An indirect effect was considered statistically significant when the 95% confidence interval did not include zero. Given the cross-sectional design, this analysis was used to examine statistical indirect effects and was not interpreted as establishing temporal or causal mediation. Statistical significance was set at *P* < .05 for all analyses.

Ethical approval was obtained from the South East Technological University Research Ethics Committee prior to commencement of the study (SETU/REC/24/25/091). Participation was voluntary and informed consent was obtained electronically before participants accessed the survey. No personally identifying information was collected, and all data were stored securely in accordance with institutional data protection procedures and the General Data Protection Regulation. Given the inclusion of questions relating to wellbeing, loneliness, and social connectedness, participants were provided with information regarding relevant university and external support services at the conclusion of the survey. The debriefing information also encouraged participants to seek informal social support where appropriate.

## Results

The final analytic sample comprised of 409 students. Participants ranged in age from 17 to 63 years (*M* = 22.26, SD = 6.94). The sample comprised students from a range of disciplines, campuses, and stages of study. Most participants identified as female (63.8%), while 32.3% identified as male and 3.9% identified as non-binary, third gender, or another gender identity. Approximately one-quarter of respondents identified as belonging to a sexual minority group (23.8%).

Students were represented across all years of study, with the largest proportion enrolled in third year (31.1%), followed by first year (27.9%) and second year (26.2%). More than half of participants reported working part-time during the academic year (54.7%), and almost half indicated that commuting affected their participation in university life (44.7%). Additional demographic and contextual characteristics are presented in Table 2.

**Table 2:** Participant characteristics (*N* = 409).

Characteristic	*N*	%
Age (years)		
Mean (SD)	22.26 (6.94)	
Range	17–63	
Gender		
Female	261	63.8
Male	132	32.3
Non-binary/third gender	11	2.7
Other	5	1.2
Sexual orientation		
Heterosexual	311	76.2
Bisexual	40	9.8
Lesbian	12	2.9
Gay	5	1.2
Pansexual	12	2.9
Queer	5	1.2
Asexual	6	1.5
Prefer not to say	16	3.9
Other	1	0.2
Ethnicity		
White Irish	283	69.5
White other background	77	18.9
Black/Black Irish	22	5.4
Asian/Asian Irish	13	3.2
Mixed race/other	11	2.7
Irish traveller	1	0.2
International student		
No	355	87.4
Yes	51	12.6
First language		
English	330	80.7
Other language	68	16.6
Multi-lingual	9	2.2
Irish	2	0.5
Disability/long-term condition		
No	283	69.4
Yes	81	19.9
Prefer not to say	31	7.6
Other	13	3.2
Study year		
First year	114	27.9
Second year	107	26.2
Third year	127	31.1
Fourth year	52	12.7
Post-graduate	9	2.2
Discipline		
Social care/social science	96	23.5
Business and law	82	20.0
Science and computing	68	16.6
Arts and creative disciplines	53	13.0
Health and sports science	48	11.7
Engineering/built environment	36	8.8
Other	20	4.9
Education	4	1.0
Campus		
Campus 1	239	58.6
Campus 2	106	26.0
Campus 3	41	10.0
Campus 4	22	5.4
Financial support		
SUSI grant	213	52.1
No support	167	40.8
BTEA	11	2.7
Scholarship	9	2.2
Student assistance fund	9	2.2
Employment status		
Not working	172	42.2
Part-time	223	54.7
Full-time	13	3.2
Hours worked per week		
None	282	68.9
1–9 h	16	3.9
10–19 h	60	14.7
20–29 h	39	9.5
30+ h	12	2.9
Travel mode to campus		
Car	157	38.5
Public transport	114	27.9
Walk	95	23.3
Lift	36	8.8
Cycle	6	1.5
Commute duration		
Under 15 min	94	23.2
15–30 min	115	28.3
30–60 min	111	27.3
Over 60 min	86	21.2
Commute affects participation		
Yes	181	44.7
No	224	55.3

### Wellbeing, belonging, loneliness, and social connectedness

Descriptive statistics for mental wellbeing, university belonging, loneliness, and social connectedness are presented in Table 3. Students reported a mean mental wellbeing score of 41.74 (SD = 9.66), a mean university belonging score of 58.32 (SD = 14.81), a mean loneliness score of 5.66 (SD = 2.09), and a mean social connectedness score of 34.13 (SD = 7.71). Mental wellbeing scores indicated that a substantial proportion of students were experiencing wellbeing difficulties. Based on established categorization thresholds, 44.1% of participants were classified as having low mental wellbeing, while 53.4% reported average wellbeing and only 2.6% reported high wellbeing.

**Table 3:** Wellbeing, belonging, loneliness and social connectedness.

Measure	*N*	Mean (SD)	Range	Variable	*N* (%)	Variable	*N* (%)
Mental wellbeing (WEMWBS)	382	41.74 (9.66)	0–70	Mental wellbeing		Loneliness	
University Belonging	357	58.32 (14.81)	0–89	Low wellbeing	171 (44.1)	Lower loneliness	156 (44.6)
loneliness (UCLA)	350	5.66 (2.09)	0–9	Average wellbeing	207 (53.4)	Higher loneliness	194 (55.4)
Social connectedness	314	34.13 (7.71)	8–50	High wellbeing	10 (2.6)		

Loneliness was also prevalent within the sample. More than half of respondents (55.4%) were classified as experiencing higher levels of loneliness, compared with 44.6% classified as experiencing lower levels of loneliness.

Students were also asked how their sense of belonging or connection to university life had changed over time. Among those who responded (*n* = 258) 37.2% reported that their sense of belonging had stayed the same, 25.6% reported that it had increased somewhat, and 7.4% reported that it had increased substantially. However, 16.3% reported that their sense of belonging had decreased somewhat, and 13.6% reported that it had decreased significantly, suggesting that a notable proportion of students feel less connected to university life over time.

Finally, students were asked whether they believed that wellbeing is supported at their university. Among respondents (*n* = 258), 41.9% agreed that wellbeing is supported, and 8.1% strongly agreed. However, 32.6% reported feeling neutral, while 13.6% disagreed and 3.9% strongly disagreed with this statement.

### Relationships between wellbeing, belonging, loneliness, and social connectedness

Pearson correlation coefficients examining relationships between mental wellbeing, university belonging, loneliness, and social connectedness are presented in Table 4. University belonging was positively associated with mental wellbeing (*r* = .51, *P* < .001) and social connectedness (*r* = .57, *P* < .001), indicating that students who reported stronger feelings of belonging also reported higher wellbeing and greater social connection. University belonging was negatively associated with loneliness (*r* = −.27, *P* < .001), suggesting that students who felt more connected to the university community experienced lower levels of loneliness. Loneliness demonstrated significant negative associations with both mental wellbeing (*r* = −.41, *P* < .001) and social connectedness (*r* = −.57, *P* < .001). The strongest relationship observed was between loneliness and social connectedness, indicating that students who felt less connected to others were more likely to experience loneliness.

**Table 4:** Correlations across mental wellbeing, university belonging, loneliness and social connectedness.

Variable	1	2	3	4
1. Mental wellbeing	–			
2. University belonging	.51***	–		
3. Loneliness	−.41***	−.27***	–	
4. Social connectedness	.45***	.57***	−.57***	–

***P < .001.

Mental wellbeing was positively associated with social connectedness (*r* = .45, *P* < .001), suggesting that students with stronger social relationships and feelings of connection reported higher levels of wellbeing. Overall, the findings indicate that wellbeing, belonging, loneliness, and social connectedness are closely interrelated, with stronger social connection and belonging associated with more positive wellbeing outcomes.

### Differences in wellbeing, belonging, loneliness, and social connectedness across student groups

Significant differences in wellbeing, loneliness, and social connectedness were observed across several demographic and contextual characteristics (Table 4). Male students reported higher levels of mental wellbeing than female students, while students identifying as part of a sexual minority group reported lower wellbeing than their heterosexual peers. Differences were particularly evident for loneliness. Female students, sexual minority students, first-year students, and students who reported that commuting negatively affected their participation in university life all reported significantly higher levels of loneliness. Campus-level differences were also observed, with loneliness score varying significantly across campuses. Significant differences in social connectedness were identified across years of study. Fourth-year students reported greater social connectedness than first-year students, suggesting that feelings of connection may strengthen over time as students become more integrated into university life.

No significant differences were observed for university belonging across demographic or contextual characteristics (gender, sexual orientation, study year, campus, disability, ethnicity, financial support, employment, etc). Similarly, wellbeing, loneliness, and social connectedness did not differ significantly according to ethnicity, international student status, first language, disability status, financial support, employment status, hours worked, travel mode, or commute duration (Table 5).

**Table 5:** Significant differences in wellbeing, belonging, loneliness, and social connectedness across student groups.

Outcome	Comparison	Group means (SD)	Mean difference	*P*-value
Mental wellbeing	Male vs female	44.56 (9.91) vs 40.29 (9.24)	4.27	<.001
	Heterosexual vs sexual minority	42.74 (9.54) vs 38.57 (9.43)	4.17	<.001
Loneliness	Female vs male	5.91 (2.09) vs 5.24 (1.97)	0.68	.027
	Sexual minority vs heterosexual	6.06 (2.23) vs 5.53 (2.03)	0.53	.043
	First year vs second year	6.25 (1.93) vs 5.14 (2.07)	1.11	.003
	Commute affects Participation vs no impact	5.99 (2.05) vs 5.39 (2.08)	0.60	.008
	Campus 2 vs campus 1^a^	6.25 (1.95) vs 5.40 (2.06)	0.85	.013
Social connectedness	Fourth year vs first year	37.38 (7.10) vs 32.04 (7.44)	5.34	.004

^a^Overall ANOVA significant across campuses; comparison shown between highest and lowest mean scores for descriptive purposes.

### Predictors of mental wellbeing

A hierarchical multiple regression analysis was conducted to examine predictors of mental wellbeing (Table 6). Demographic variables (gender, sexual orientation, and study year) were entered in the first step, perceived effects of commuting were entered in the second step, and psychosocial variables (university belonging, loneliness, and social connectedness) were entered in the final step. The initial model, containing demographic variables, explained 10.5% of the variance in mental wellbeing (*R*^2^ = .105, *P* < .001). The addition of commuting in Step 2 resulted in a small increase in explained variance (*R*^2^ = .112), although commuting was not a significant independent predictor of wellbeing. The inclusion of psychosocial variables in the final model substantially improved model fit, increasing the explained variance to 41.7% (*R*^2^ = .417, *P* < .001). In the final model, university belonging emerged as the strongest positive predictor of mental wellbeing (*β* = .325, *P* < .001). Loneliness was a significant negative predictor (*β* = −.207, *P* < .001), while social connectedness was positively associated with wellbeing (*β* = .150, *P* = .015). Gender (*β* = .162, *P* < .001), sexual orientation (*β* = −.188, *P* < .001), and study year (*β* = −.145, *P* = .001) also remained significant predictors. In contrast, the perceived effects of commuting were not independently associated with wellbeing after psychosocial factors were included in the model. Overall, the findings indicate that psychosocial factors accounted for a substantial proportion of variation in student wellbeing, with university belonging emerging as the most influential predictor.

**Table 6:** Hierarchical regression predicating mental wellbeing.

Predictor	*β*	*P*
Gender	.162	<.001
Sexual orientation	−.188	<.001
Study year	−.145	.001
Commute impact	.022	.621
University belonging	.325	<.001
Loneliness	−.207	<.001
Social connectedness	.150	.015
**Model statistics**	***R* ^2^**	
Step 1: Demographics	.105	
Step 2: +Commute impact	.112	
Step 3: +Belonging, loneliness, connectedness	.417	

Final model: *F*(7304) = 31.04, *P* < .001.

### Mediation analysis

Finally, a mediation analysis was conducted to examine the indirect association between loneliness and mental wellbeing through university belonging. Loneliness was significantly negatively associated with university belonging (*b* = −2.16, SE = 0.35, *P* < .001), indicating that students reporting higher levels of loneliness also reported lower levels of belonging. When loneliness and belonging were entered simultaneously as predictors of wellbeing, both variables remained significant. Higher levels of belonging were associated with better mental wellbeing (*b* = 0.27, SE = 0.03, *P* < .001), while higher loneliness was associated with poorer wellbeing (*b* = −1.23, SE = 0.20, *P* < .001). The model explained 33.2% of the variance in wellbeing (*R*^2^ = .332, *P* < .001). The total association between loneliness and wellbeing was significant (*b* = −1.81, SE = 0.21, *P* < .001). The indirect effect of loneliness on wellbeing through university belonging was also significant (*b* = −0.58, 95% CI [−0.95, −0.28]). As the confidence interval did not include zero, the findings were statistically consistent with an indirect association involving university belonging. Results indicate that loneliness was associated with poorer wellbeing both directly and indirectly through lower levels of university belonging. Students who reported greater loneliness were less likely to feel connected to the university community, with lower levels of belonging also associated with lower mental wellbeing (Fig. 1).

**Figure 1:**
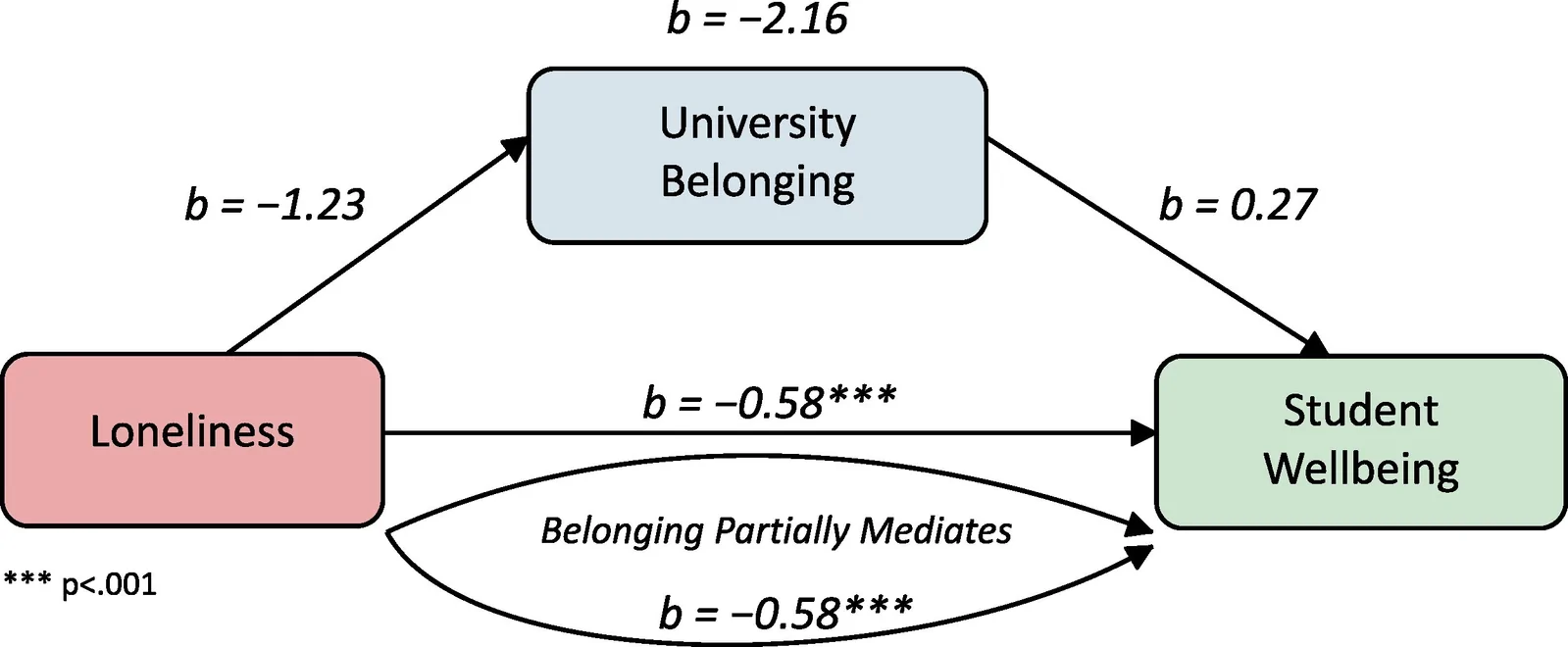
Mediation model examining the association between loneliness, university belonging, and mental wellbeing.

## Discussion

This study examined wellbeing, belonging, loneliness, and social connectedness among students attending a multi-campus technological university in Ireland through a settings-based health promotion lens where a number of important findings were evident. Substantial proportions of students reported low mental wellbeing and elevated loneliness, reflecting growing concerns regarding social disconnection and psychological wellbeing among young adults and wider populations (Lim *et al*. 2020, Shah and Househ 2023, WHO 2024). Increasing attention has been drawn to loneliness, declining social participation, weakening community ties, and reductions in opportunities for meaningful social connection at a societal level (Putnam 2000, Holt-Lunstad 2024). From a mental health promotion perspective, these findings should not be understood solely as individual difficulties, but as experiences shaped by the wider social, economic, cultural, and environmental conditions in which students live, learn, and participate (Barry 2009). Universities are not immune from these broader societal trends and represent an important everyday setting with the capacity to actively foster connection, participation, and community among diverse populations (HEA 2021, Dooley *et al*. 2026).

Wellbeing, belonging, loneliness, and social connectedness were also closely interrelated, highlighting the importance of students’ social experiences for mental wellbeing. Most notably, university belonging emerged as the strongest predictor of wellbeing, explaining variance beyond demographic and contextual characteristics. Belonging was positively associated with wellbeing and social connectedness, negatively associated with loneliness, and remained the strongest predictor of wellbeing after accounting for demographic characteristics, commuting-related factors, and other psychosocial variables. A statistically significant indirect association between loneliness and wellbeing through university belonging was also observed. Although the cross-sectional design does not allow temporal ordering or causal mediation to be established, the findings are consistent with a model in which belonging may play an intermediary role in the relationship between loneliness and wellbeing. Students reporting greater loneliness also tended to report lower university belonging, while stronger belonging was associated with better mental wellbeing. These findings suggest that belonging represents a central psychosocial resource within higher education (Thomas 2012, Strayhorn 2019, Allen *et al*. 2021). Importantly, alternative or reciprocal relationships between loneliness, belonging, and wellbeing are also plausible. For example, stronger mental wellbeing may facilitate participation and social integration within university life, creating greater opportunities to develop belonging, while poorer wellbeing may constrain students’ capacity to engage with such opportunities. Equally, loneliness may contribute to how connected students perceive themselves to be within the university community. The present cross-sectional data cannot distinguish between these possible directions of association, and longitudinal research is needed to examine their temporal ordering and potentially reciprocal relationships.

In addition, the present findings extend emerging evidence from Irish university populations, highlighting the importance of social experiences for student mental health. Previous research among male university students identified loneliness as one of the strongest predictors of suicide risk, while resilience appeared protective against psychological distress. Loneliness remained independently associated with suicide risk even after accounting for behavioural, demographic, and masculinity-related factors (O'Donnell *et al*. 2025, McGrath *et al*. 2026). Similarly, attitudes towards help-seeking and psychological wellbeing appear closely intertwined with students’ social experiences and perceptions of connection within the university environment (Thomas 2012, Strayhorn 2019, Pedler *et al*. 2022). These studies suggest that social experiences occupy a central position within the mental health continuum of university students. Importantly, belonging may also represent a broader and more positive construct than simply the absence of loneliness. While loneliness reflects perceived social isolation, belonging captures whether students feel accepted, valued, included, and recognized as legitimate members of the university community (Thomas 2012). The finding that belonging emerged as the strongest predictor of wellbeing suggests that promoting student wellbeing requires more than reducing distress or responding to mental health difficulties. Consistent with settings-based health promotion approaches, it also requires creating university environments that actively foster participation, inclusion, meaningful connection and a sense of community, enabling students to feel valued, supported and connected within university life (WHO 1986, 2015, Dooris and Doherty 2010, HEA 2025, Dooley *et al*. 2026). Notably, no significant differences in belonging were observed across demographic or contextual groups, despite differences in wellbeing and loneliness. This suggests that belonging may function as a broadly relevant protective resource across the student population (Strayhorn 2019, Allen *et al*. 2021). Regardless of background or circumstance, students who reported stronger feelings of belonging also reported better wellbeing.

However, while belonging appeared universally beneficial, opportunities to develop belonging may not be universally available (Vaccaro and Newman 2016). Significant differences in wellbeing, loneliness, and social connectedness were observed across several groups, including first-year students, sexual minority students, and students whose commuting affected participation in university life. These findings suggest that some students may face greater challenges in accessing the social experiences and opportunities through which belonging is cultivated (Tett *et al*. 2025). The findings may also support the view that belonging is developmental rather than immediate. First-year students reported significantly higher loneliness and lower social connectedness than students in later years, while fourth-year students reported the highest levels of social connectedness. Although belonging scores themselves did not differ significantly across years of study, one possible interpretation is that the social foundations upon which belonging is built may strengthen over time as students develop relationships, become familiar with institutional cultures, and establish a sense of place within university life. This aligns with evidence identifying transition into higher education as a period of heightened vulnerability to social isolation and psychological distress (Worsley *et al*. 2021). Indeed, these findings highlight an important tension within contemporary higher education. Although belonging may be universally beneficial, it may also operate as a form of social resource that is unevenly distributed (Vaccaro and Newman 2016, Li *et al*. 2025, Tett *et al*. 2025) This interpretation is consistent with recent Irish work on student engagement and belonging, which highlights how commuting, housing pressures, financial precarity, and limited access to social spaces can constrain students’ capacity to participate in university life (Dooley *et al*. 2026). Therefore, students who experience barriers to participation, whether related to social identity, life circumstances, or structural constraints, may have fewer opportunities to access the relationships, activities, and communities through which belonging develops. From this perspective, belonging should be understood beyond an individual psychological state and also as something produced through social relationships, institutional practices, and access to participation. This perspective also aligns with the Limerick Framework for Action, particularly its emphasis on People and Places and on creating health-promoting campus environments through whole-system approaches that enable participation, inclusion and wellbeing (International Health Promoting Campuses Network and University of Limerick 2025).

The findings have important implications for Healthy Campus and Health Promoting University approaches. Traditionally, student wellbeing initiatives have often focused on the provision of counselling, mental health supports and individual behaviour change. While these services remain important, the present findings, alongside a growing body of research, support the view that wellbeing is also shaped by broader social, organizational and environmental conditions that influence opportunities for connection, participation and belonging (WHO 1986, 2015, Dooris and Doherty 2010, HEA 2021). This suggests that promoting student wellbeing requires not only responding to distress but also creating university environments that actively support inclusion, community and meaningful social connection. Consistent with the Ottawa Charter (WHO 1986) and Okanagan Charter (WHO 2015), universities should therefore view belonging as a health promotion priority rather than solely a student engagement outcome. Indeed, efforts to promote wellbeing may benefit from greater attention to the social architecture of university life, including opportunities for participation, peer connection, inclusive campus cultures, curriculum design, transition supports, and institutional practices that foster inclusion and community. The significant differences in loneliness observed across campuses are interesting in this regard. While the reasons cannot be determined from the present study, it may indicate that experiences of connection and isolation may vary across institutional settings and are likely influenced by local social, environmental, and organizational factors (Dooris and Doherty 2010, Thomas 2012, WHO 2015). This reinforces the importance of viewing universities not as single, homogeneous environments but as collections of interconnected settings, each with the potential to either facilitate or constrain opportunities for belonging (HEA 2021).

The findings are particularly relevant for increasingly diverse higher education populations. This aligns with the Irish National Access Plan 2022–2028, which moves beyond access alone to emphasize equity of participation, success, and student experience within increasingly diverse higher education populations (HEA 2022). In the present study, poorer outcomes were observed among selected groups, including first-year students, sexual minority students, and students whose commuting affected their participation in university life. These findings suggest that the conditions through which belonging develop may not be equally accessible to all students (Verbree *et al*. 2025). As universities welcome greater numbers of mature students, commuting students, international students, and students from historically under-represented groups, creating equitable opportunities for connection becomes increasingly important. A Healthy Campus approach therefore requires not only supporting individual students experiencing difficulties but also creating institutional environments in which belonging can flourish across the student population. Belonging may be universally beneficial, but universities have a critical role in ensuring it is also universally accessible.

## Strengths and limitations

This study has several strengths. It draws on data from a large and diverse sample of students across multiple campuses, disciplines, and stages of study within a higher education institution. This enabled the examination of wellbeing, belonging, loneliness, and social connectedness across a broad range of student experiences. The study also employed validated measures of wellbeing, belonging, loneliness, and social connectedness, allowing comparison with existing national and international research. By examining these constructs simultaneously, the study was able to explore the relative contribution of belonging, loneliness, and social connectedness to student wellbeing and examine the pattern of associations between these experiences. The study also contributes to the growing literature on Health Promoting Universities by examining belonging through a settings-based health promotion lens. While belonging is frequently discussed within student engagement and retention literature, it has received comparatively less attention as a health-promoting resource with implications for wellbeing. The inclusion of indirect-effect analysis further enabled examination of whether the association between loneliness and wellbeing included a statistically significant indirect association through university belonging.

Several limitations should also be acknowledged. The cross-sectional design limits the ability to draw causal inferences regarding the relationships observed. This is particularly important when interpreting the indirect-effect analysis. Although a statistically significant indirect association between loneliness and wellbeing through university belonging was observed, the concurrent measurement of all variables means that temporal ordering and causal mediation cannot be established and alternative and potentially reciprocal relationships are also plausible. Longitudinal research is needed to examine the temporal ordering and potentially bidirectional relationships between belonging, loneliness, and wellbeing over time. The use of convenience sampling may also have introduced self-selection bias. The study was conducted within a single higher education institution and relied on self-reported survey data, which may limit the generalizability of findings to other institutional contexts. In addition, the sample contained a higher proportion of female participants and students from particular campuses and disciplines, which may have influenced the findings. Finally, while the study identified significant differences across selected student groups, it was not designed to examine the specific mechanisms through which belonging is fostered or constrained. Future research should explore how institutional practices, learning environments, and opportunities for participation shape students’ experiences of belonging across diverse higher education contexts.

## Conclusion

This study highlights the central role of belonging in shaping student wellbeing within higher education. While substantial proportions of students reported low mental wellbeing and elevated loneliness, university belonging emerged as the strongest predictor of wellbeing. These findings suggest that belonging represents more than a desirable aspect of the student experience and it may function as an important health-promoting resource associated with positive mental wellbeing. The findings also indicate that opportunities for connection and participation may not be experienced equally across the student population. First-year students, sexual minority students, and students whose commuting affected participation reported poorer outcomes on selected measures, highlighting the importance of considering how social, organizational, and environmental conditions shape access to belonging. Belonging may be universally beneficial, but it is not necessarily universally accessible. From a health promotion perspective, the findings reinforce the importance of moving beyond individualized approaches to student wellbeing and towards whole-university strategies that create the conditions for connection, participation, inclusion, and community. Consistent with Healthy Campus and Health Promoting University frameworks, universities have the potential to influence wellbeing not only through support services, but through the everyday environments, relationships, and institutional practices that shape whether students feel that they belong. As higher education institutions become increasingly diverse and continue to respond to growing concerns regarding student mental health and social disconnection, fostering belonging should be recognized as both a student success priority and a health promotion imperative.

## Data Availability

The data underlying this article are not publicly available due to ethical and privacy considerations, as participants did not provide consent for public data sharing and the dataset contains potentially identifiable information. De-identified data may be made available from the corresponding author upon reasonable request, subject to approval by the South East Technological University Research Ethics Committee and in accordance with applicable data protection and ethical requirements.
